# Regulation of soluble CD127 protein release and corresponding transcripts expression in T lymphocytes from septic shock patients

**DOI:** 10.1186/s40635-018-0220-3

**Published:** 2019-01-08

**Authors:** Julie Mouillaux, Camille Allam, Thomas Rimmelé, Thomas Uberti, Benjamin Delwarde, Julien Textoris, Guillaume Monneret, Estelle Peronnet, Fabienne Venet

**Affiliations:** 1 EA 7426 « Pathophysiology of injury-induced immunosuppression (PI3) » Lyon 1 University / Hospices Civils de Lyon / bioMérieux, 5 place d’Arsonval, 69437 Lyon Cedex 03, France; 2 Joint Research Unit HCL-bioMérieux-Lyon 1 University, Hôpital Edouard Herriot, 5 place d’Arsonval, 69003 Lyon, France; 3 Immunology Laboratory, Hospices Civils de Lyon, Hôpital Edouard Herriot, 5 place d’Arsonval, 69003 Lyon, France; 4 Anesthesiology and Intensive care medicine department, Hospices Civils de Lyon, Hôpital Edouard Herriot, 5 place d’Arsonval, 69003 Lyon, France

To the Editor,

Sepsis is the leading cause of death for critically ill patients. Septic patients develop T lymphocyte dysfunctions associated with increased mortality and nosocomial infections [1]. Therefore, IL-7 has been recently evaluated in a clinical trial to reverse these alterations [2]. IL-7 receptor (IL-7R) is composed of an IL-7-specific chain (CD127) and a common receptor γ-chain [3]. IL-7R exists in a soluble form (sCD127) [4], resulting from shedding from the cell surface or transcriptional regulation [5, 6]. Several transcripts missing the exon 6, coding for the transmembrane domain have been identified, such as IL7R3 [4, 6] and IL7R7 (Ensembl). We recently showed that plasmatic sCD127 concentration and whole blood IL7R3 transcript expression were decreased in septic shock [7, 8]. However, sCD127 protein and transcripts regulations in survivor and non-survivor patients differed, with higher sCD127 concentration and lower IL7R3 expression in non-survivors. Therefore, the regulation of soluble IL-7R in sepsis remains not fully understood.

In this study, we evaluated sCD127 release and corresponding transcripts IL7R3 and IL7R7 expressions in purified T cells from septic shock patients. After approval by local ethics committee, T cells were isolated from 32 septic shock patients (Table 1) and 31 healthy volunteers (HV) (Additional file 1).

**Table 1:** Clinical characteristics of septic shock patients

Parameters	Septic shock patients (*n* = 32)
Sex, male	26 (68%)
Age, years	70 [62–76]
SAPS II score	61 [51–82]
SOFA score (*n* = 31)	9 [8–12]
Charlson co-morbidity score (*n* = 30)	
0	3 (10%)
1	14 (47%)
> 1	13 (43%)
Mac Cabe score (*n* = 31)	
Non-fatal diseases	21 (68%)
Ultimately fatal diseases	9 (29%)
Rapidly fatal diseases	1 (3%)
Initial infection	
Abdominal infection	16 (50%)
Pneumopathy	4 (12.5%)
Urinary infection	4 (12.5%)
Other	8 (25%)
Type of admission	
Medical	13 (41%)
Emergency surgery	18 (56%)
Elective surgery	1 (3%)
Microbiological documentation	
Gram negative	10 (31%)
Gram positive	7 (22%)
Other	1 (3%)
Non-documented	14 (44%)
Mortality at D28	9 (28%)
Lactate at D1 (mmol/L)	2.8 [1.7–5.6]
Lymphocytes at D3 (10^9^/L)	1.05 [0.6–1.35]
mHLA-DR at D3 (AB/C)	8199 [3076–12,171]

For clinical parameters, categorial data are presented as numbers of cases and percentages of the total population in brackets. Continuous data and biological parameters are presented as medians and interquartile ranges [Q1-Q3]. Simplified Acute Physiology Score II (SAPS II) and Mac Cabe score were calculated on admission. Sequential Organ Failure Assessment (SOFA) score was measured after 24 h of intensive care unit stay. mHLA-DR (AB/C): number of anti-HLA-DR antibodies bound per monocyte

Interestingly, the sCD127 release tended to be higher in patients’ T cells supernatant compared to HV (Fig. 1). This contrasts with the decreased plasmatic sCD127 concentration in septic shock patients [7], possibly impacted by lymphopenia. Both IL7R3 and IL7R7 transcripts expressions were decreased in septic shock patients’ T cells (Fig. 2), as we previously observed in whole blood [8]. While whole blood IL7R3 and IL7R7 expressions may be impacted by sepsis-induced lymphopenia, these transcripts are also intrinsically regulated in T cells.

**Fig. 1:**
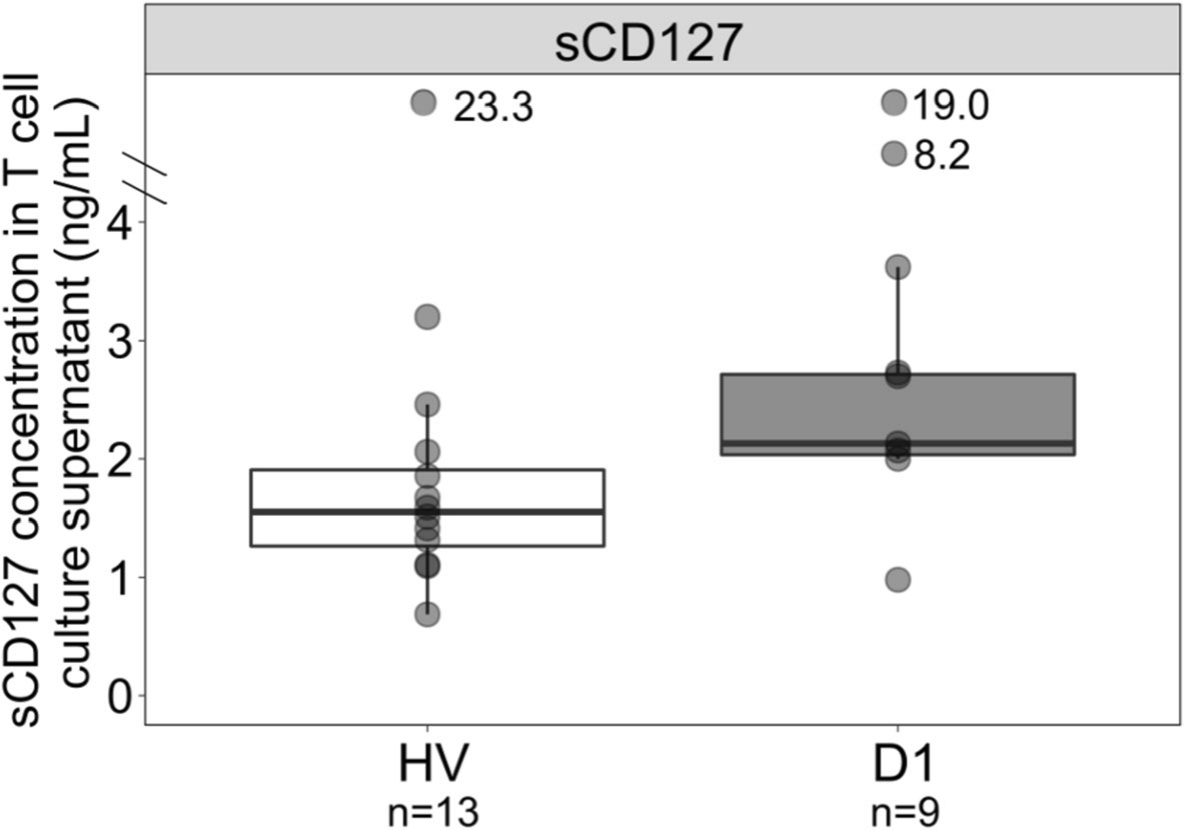
sCD127 protein release from purified T cells from septic shock patients. sCD127 release was quantified in supernatants of purified T cells of septic shock patients (D1, *n* = 9) in comparison with healthy volunteers (HV, *n* = 13) after 48 h of culture without any stimulation. Data are presented as Tukey boxplots. Mann-Whitney tests were used to compare values between septic shock patients and HV. **p < 0.01, ***p < 0.001. See Additional file 1 for details

**Fig. 2:**
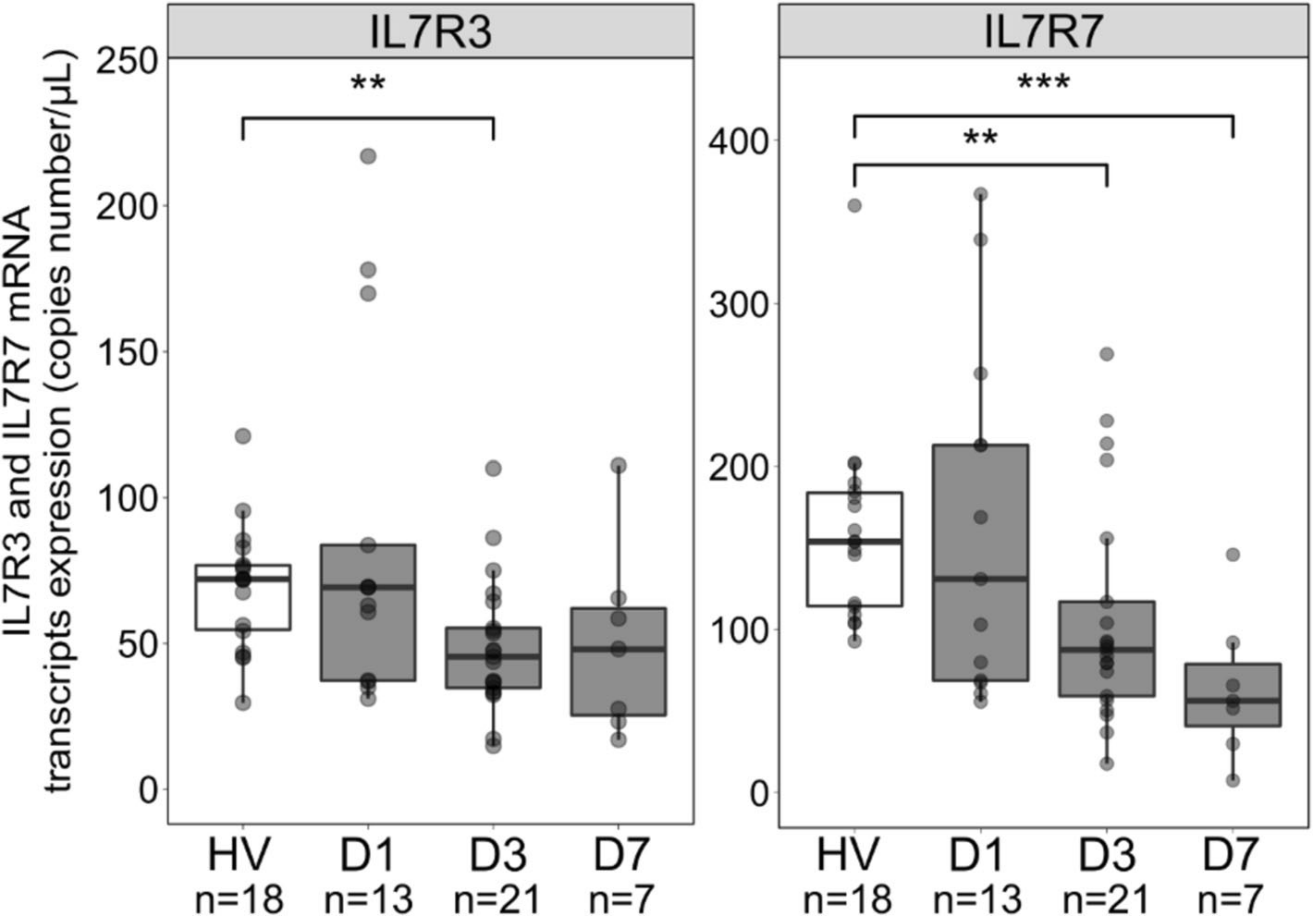
IL7R mRNA transcripts expression in purified T cells from septic shock patients. Gene expressions of the IL7R3 and IL7R7 transcripts were measured using RT-qPCR from RNA from purified T cells from septic shock patients at D1 (*n* = 13), D3 (*n* = 21), and D7 (*n* = 7) in comparison with HV (*n* = 18). Data are presented as Tukey boxplots. Mann-Whitney tests were used to compare values between septic shock patients and HV. **p < 0.01, ***p < 0.001. See Additional file 1 for details

To delineate soluble IL-7R regulation in sepsis, we aimed to reproduce ex vivo its expression pattern observed in patients, by stimulating T cells from HV with IL-7 or TCR activation, known to regulate CD127 expression [9]. sCD127 was spontaneously released from non-stimulated T cells (Fig. 3a). During IL-7 stimulation, sCD127 protein release and transcripts expression decreased. In contrast, TCR activation induced an opposite regulation of sCD127 and corresponding transcripts (Fig. 3b): sCD127 concentration increased, as previously described [10], while IL7R7 and IL7R3 transcripts expressions decreased. This suggests that these transcripts are not the main source of sCD127 in this context, and that other mechanisms, such as shedding of membrane CD127, might occur. Overall, ex vivo TCR activation partly reproduced soluble IL-7R expression pattern observed in septic shock, suggesting that the initial T cell activation shown to occur in sepsis [11, 12] could participate to soluble IL-7R regulation, both at the transcriptional and protein levels.

**Fig. 3:**
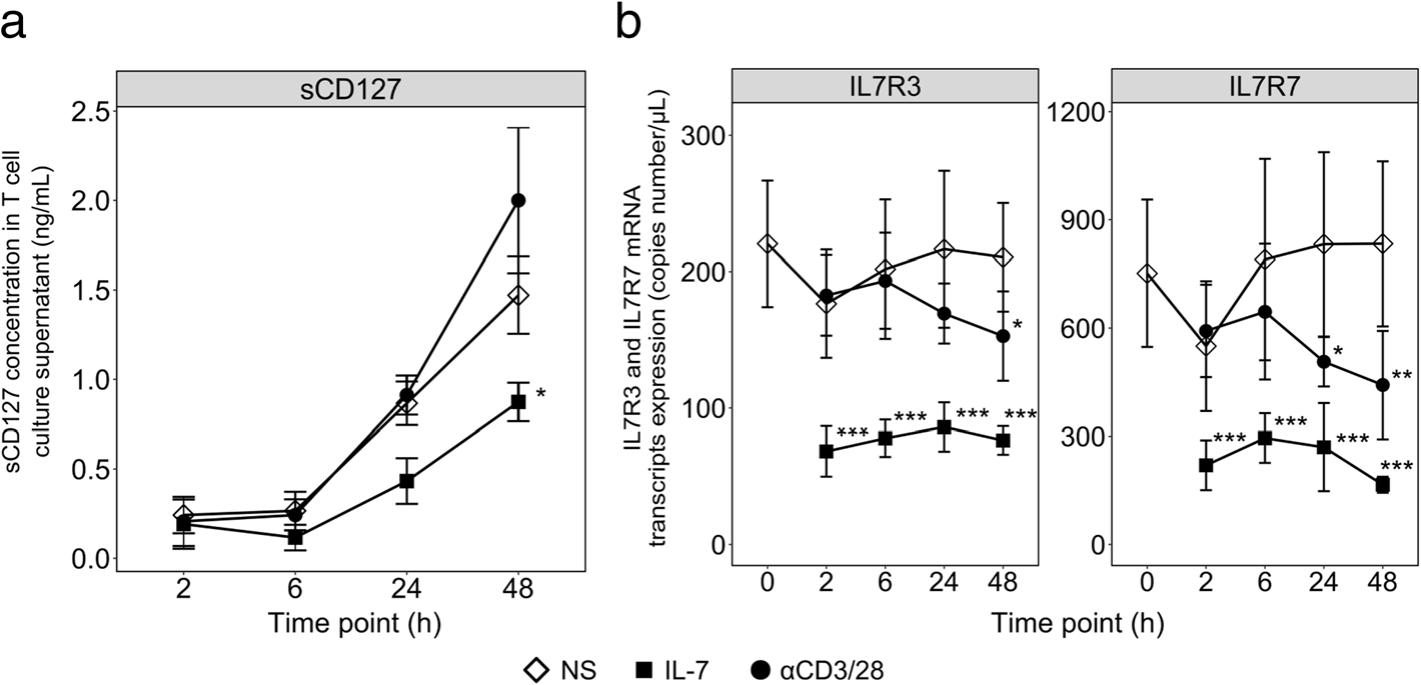
sCD127 release and transcripts expression upon ex vivo stimulation of healthy volunteers’ purified T cells. **a** sCD127 protein release and **b** IL7R3 and IL7R7 mRNA transcripts expressions were measured in purified T cells from healthy volunteers (HV) stimulated ex vivo after the indicated time of incubation with IL-7 (*n* = 5, 10 ng/mL), anti-CD3/CD28 antibodies coated beads (*n* = 5, αCD3/28, 1:2 bead to cells ratio), or not stimulated (*n* = 10, NS). For each HV and each condition of stimulation, samples were proceeded in biological triplicates (culture wells) and technical duplicates (for ELISA only), summarized by the mean of the values for each individual. Data are presented as means and standard error of the mean. Mann-Whitney tests were used to compare values between non-stimulated and stimulated purified T cells from HV. *p < 0.05, **p < 0.01, ***p < 0.001. See Additional file 1 for details

Altogether, we report here an intrinsic downregulation of IL-7R soluble transcripts in septic shock patients’ T cells, independently of lymphopenia, in parallel with an increased sCD127 protein release. Ex vivo experiments in cells from HV suggest that initial T cell activation after sepsis might participate in this regulation, although this remains to be formally demonstrated.

## Additional file

Additional file 1: Methods. (PDF 352 kb)

## Abbreviations

HV: Healthy volunteer
IL-7R: IL-7 receptor
sCD127: Soluble CD127
αCD3/28: Anti-CD3/CD28 antibodies coated beads
