# Supplementary material for: Regulation of soluble CD127 protein release and corresponding transcripts expression in T lymphocytes from septic shock patients
Source: Intensive Care Med Exp. 2019 Jan 8;7:3. doi: 10.1186/s40635-018-0220-3 (PMC6325048; doi:10.1186/s40635-018-0220-3)
Supplement: Supplementary file 1 — Methods. (PDF 352 kb) [file 40635_2018_220_MOESM1_ESM.pdf]

Additional Methods

Regulation of soluble CD127 protein release and corresponding transcripts expression in T lymphocytes from septic shock patients

Julie Mouillaux, Camille Allam, Thomas Rimmelé, Thomas Uberti, Benjamin Delwarde, Julien Textoris, Guillaume Monneret, Estelle Peronnet, Fabienne Venet

Septic shock patients and healthy volunteers

This study was conducted in the Intensive Care Units (ICU) of the Edouard Herriot hospital (Hospices Civils de Lyon, Lyon, France). This project was approved by our Institutional Review Board for ethics ("Comité de Protection des Personnes Sud-Est II"), which waived the need for informed consent, because this study was observational and performed on residual blood after completion of routine follow-up (#IRB 11236). This study is registered at the French Ministry of Research and Teaching (#DC-2008-509), at the Commission Nationale de l'Informatique et des Libertés and on clinicaltrials.gov (NCT02803346). Non-opposition to inclusion in the study was registered for each patient. Inclusion and exclusion criteria were described previously [1]. mHLA-DR (AB/C), number of anti-HLA-DR antibodies bound per monocyte, was measured as previously described [2]. Peripheral whole blood samples were collected at three time-points after the onset of septic shock: from 24 h to 48 h (D1, n=22), 72 h to 96 h (D3, n=21) and 7 to 8 days (D7, n=7).

Peripheral blood from healthy volunteers (HV) was provided by "Etablissement Français du Sang": 21 age and gender matched HV with the septic shock patients' cohort and 10 HV for the *ex vivo* experiments. Healthy volunteers were selected based on a global-based and not an individual-based matching with the septic shock patients. This explains why the number of included healthy donors is not similar to the number of included septic shock patients. According to the standardized procedure for blood donation, written informed consent was obtained from HV and personal data for blood donors were anonymized at time of blood donation and before blood transfer to our research lab.

In total, samples from 31 healthy volunteers were used in the experiments performed in this study. The repartition is as follows: 13 healthy volunteers were used for measuring sCD127 release (Figure 1) and 18 for the measuring IL-7R mRNA expression with septic shock patients (Figure 2). Ten healthy donors were common between these 2 analyses. Finally, 10 healthy volunteers were used for the ex vivo stimulation experiments (Figure 3). These were different individuals than the first two figures.

Regarding septic patients, samples from 9 septic shock patients were presented in Figure 1. None of these samples was used in Figure 2. The other 23 septic shock patients analyzed in Figure 2 were

distributed as follows: six patients had samples at D1, D3 and D7; five patients had samples at D1 and D3; one patient at samples at D3 and D7; two patients had only a D1 sample and nine patients only a D3 sample.

In the experiments measuring IL7R3 and IL7R7 mRNA expression, we verified that for six patients for whom we had samples available at the three time points, the expression profiles overtime were similar to the all cohort (See **Additional Figure 1** below). Indeed, we observed a downregulation of soluble IL7R transcripts in septic shock patients compared to healthy volunteers. IL7R7 expression was significantly decreased at D7 compared to D1. These results are in agreement with the conclusions reported in the manuscript when considering all patients.

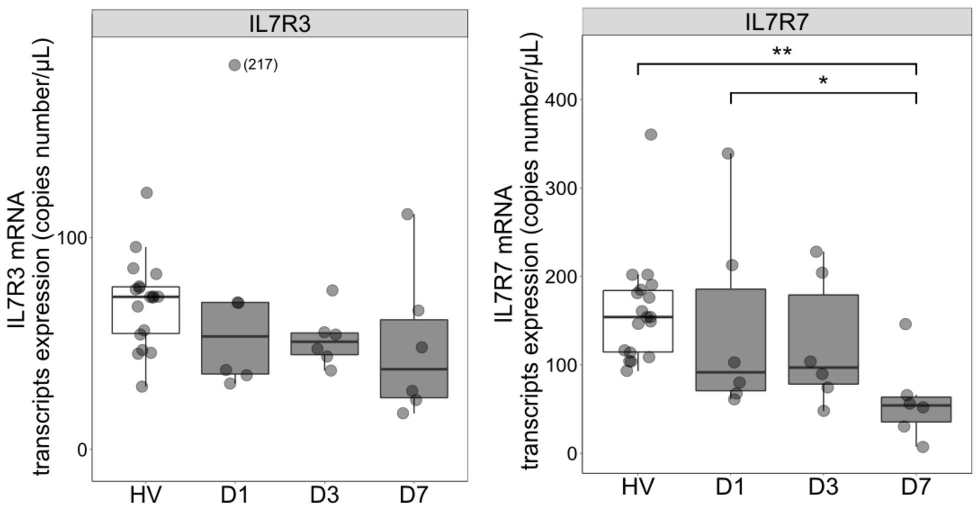

Additional Figure 1 IL7R mRNA transcripts expression in purified T cells from septic shock patients with a sample collected at the three time points. Gene expressions of the IL7R3 and IL7R7 transcripts were measured using RT-qPCR from RNA from purified T cells from septic shock patients at D1, D3 and D7 (n=6) in comparison with HV (n=18). Data are presented as Tukey boxplots. Mann-Whitney tests were used to compare values between septic shock patients and HV (**p<0.01) and paired Wilcoxon tests were used to compare values between the different time points in septic shock patients (*p<0.05).

T cell purification

Human T cells were isolated from HV and septic shock patients whole blood samples by antibody-based negative selection and density gradient centrifugation using a human T cell enrichment cocktail (Rosette Sep^{TM}, StemCell Technologies, Grenoble, France), according to the manufacturer's instructions. Quality of T cell purification was systematically controlled. Purified cells were labeled with a Pacific Blue anti-CD3 antibody (Ab) (Beckman Coulter, Hialey, FL, USA) and lithium dodecyl sulfate 751 as a marker of nucleated cells (Molecular Probes, Life Technologies, Saint Aubin, France). The sample purity was systematically higher than 95 %. Purified T cells were suspended in complete culture medium: RPMI 1640 (Eurobio, Les Ulis, France), supplemented with 10 % AB human serum (Life Technologies), 2 mM L-

Glutamine (Eurobio), 1,000 IU/mL penicillin (Eurobio), 1,000 µg/mL streptomycin (Eurobio) and 200 µg/mL amphotericin B (Gibco, Thermofisher scientific, Wilmslow, UK).

IL-7 receptor expression in response to different stimuli

The purified T cells from HV were cultured at 1×106 cells per mL in complete culture medium and incubated at 37 °C in humidified 5 % CO2 atmosphere during the indicated time (2, 6, 24 and 48 h). Triplicates were performed for each culture condition. Two stimuli were used: anti-CD3/CD28 antibodies coated beads (αCD3/28) (T cell activation/expansion kit, 1:2 bead to cells ratio according to the manufacturer's instructions, Miltenyi Biotec, Aubrun, CA, USA) and IL-7 (10 ng/mL, R&D systems, Minneapolis, MN, USA). IL-7 concentration was selected from previous studies reporting an effect of IL-7 on its own receptor expression ex vivo [3–5].

IL7R3 and IL7R7 transcript expression measurement

Total RNA was extracted using an RNeasy kit (Qiagen, Hilden, Germany) from T cells, immediately after purification for septic shock patients and HV samples, or after the indicated time of culture with the different stimuli. RNA integrity was assessed with the RNA 6000 Nano kit (Agilent technologies, Santa Clara, CA, USA). Total RNA was reversed transcribed in complementary DNA (cDNA) using SuperScript®VILO™ cDNA synthesis kit (Life Technologies). Specific PCR assays were designed to quantify two IL7R transcripts expressions: IL7R3 coding for sCD127 and IL7R7 potentially coding for sCD127 (**Additional Table S1**). Specificity of PCR assays was confirmed by amplicon sequencing. Quantitative PCR was conducted on a LightCycler 480 instrument (Roche, Bale, Switzerland) using LightCycler 480 probes master kit (Roche) as previously described [6]. Results were expressed as absolute concentrations and normalized by the RNA quantity used for the reverse transcription (100 ng for septic shock patients purified T cells and 200 ng for *ex vivo* cultured purified T cells).

Additional Table S1 PCR assays characteristics

**PCR design**	**Primers and probes sequences**		**Target exon**	**Product length (bp)**
**IL7R3**	Forward	GCTCAGGATTAAGCCTATCG	5-7	131
	Backward	CACTGGGATGTTGCCAACAC	7	
	Probe	ATCATAAGAAGACTCTGGAACATCT	7	
**IL7R7**	Forward	GGAAGTGAATGGATCGCAGC	2	313
	Backward	CAGAATGTCCAGACACAGTG	3	
	Probe	CTGTGCTTTTGAGGACCCAGAT	2	

IL7R mRNA transcripts were named according to Ensembl release 87.

Soluble CD127 measurement

sCD127 was measured in culture supernatant of purified T cells from septic shock patients and HV after 48 h of culture without any stimulation, and in supernatant of purified T cells from HV with *ex vivo* stimulation after the indicated time of culture (2, 6, 24 and 48 h) using a sandwich ELISA, adapted from Peronnet *et al.* [7]. Recombinant human CD127 Fc chimera protein (R&D Systems), used as standard protein, was diluted in RPMI1640 (Eurobio) with 10 % AB human serum (Life Technologies). The detection limit was 0.898 ng/mL.

Statistics

Results are presented as means and standard error of the mean or as Tukey boxplots. Bottom and top of the box represent the first and third quartiles, respectively. The horizontal bar within the box represents the median value. Lower and higher extremities of the whiskers respectively represent the lowest datum still within 1.5 inter-quartile range of the lower quartile, and the highest datum still within 1.5 inter-quartile range of the upper quartile. Mann-Whitney tests were used to assess variations between septic shock patients and HV and between *ex vivo* non-stimulated and stimulated T cells purified from HV. Statistical analyses were performed using R statistics software (R version 3.2.4). P-values lower than 0.05 were considered statistically significant.

Additional References

1. Demaret J, Villars-Méchin A, Lepape A, et al (2014) Elevated plasmatic level of soluble IL-7 receptor is associated with increased mortality in septic shock patients. Intensive Care Med 40:1089–1096. doi.org/10.1007/s00134-014-3346-0

2. Demaret J, Walencik A, Jacob M-C, et al (2013) Inter-laboratory assessment of flow cytometric monocyte HLA-DR expression in clinical samples. Cytometry B Clin Cytom 84:59–62. doi.org/10.1002/cyto.b.21043

3. Paiardini M, Cervasi B, Albrecht H, et al (2005) Loss of CD127 Expression Defines an Expansion of Effector CD8+ T Cells in HIV-Infected Individuals. J Immunol 174:2900–2909. doi.org/10.4049/jimmunol.174.5.2900

4. Monti P, Brigatti C, Krasmann M, et al (2013) Concentration and Activity of the Soluble Form of the Interleukin-7 Receptor α in Type 1 Diabetes Identifies an Interplay Between Hyperglycemia and Immune Function. Diabetes 62:2500–2508. doi.org/10.2337/db12-1726

5. Alves NL, Leeuwen EMM van, Derks IAM, Lier RAW van (2008) Differential Regulation of Human IL-7 Receptor α Expression by IL-7 and TCR Signaling. J Immunol 180:5201–5210. doi.org/10.4049/jimmunol.180.8.5201

6. Delwarde B, Peronnet E, Venet F, et al (2018) Low Interleukin-7 Receptor Messenger RNA Expression Is Independently Associated With Day 28 Mortality in Septic Shock Patients. Crit Care Med. doi.org/10.1097/CCM.0000000000003281

7. Peronnet E, Mouillaux J, Monneret G, et al (2016) Elevated soluble IL-7 receptor concentration in non-survivor ICU patients. Intensive Care Med 42:1639–1640. doi.org/10.1007/s00134-016-4445-x
